# Gingiva-Derived Mesenchymal Stem Cells Attenuate Imiquimod- (IMQ-) Induced Murine Psoriasis-Like Skin Inflammation

**DOI:** 10.1155/2022/6544514

**Published:** 2022-06-30

**Authors:** Ziyu Ye, Yanfang Liang, Bihua Lin, Yanyun Li, Xingxing Chai, Jiachun Lian, Xueying Zhang, Zhengping Che, Jincheng Zeng

**Affiliations:** ^1^Dongguan Key Laboratory of Medical Bioactive Molecular Developmental and Translational Research, Guangdong Provincial Key Laboratory of Medical Molecular Diagnostics, Guangdong Medical University, Dongguan 523808, China; ^2^Guangdong Xinghai Institute of Cell, Dongguan 523808, China; ^3^Department of Pathology, Dongguan Hospital Affiliated to Jinan University, Binhaiwan Central Hospital of Dongguan, Dongguan 523000, China; ^4^Key Laboratory of Medical Bioactive Molecular Research for Department of Education of Guangdong Province, School of Basic Medicine, Guangdong Medical University, Dongguan 523808, China; ^5^Collaborative Innovation Center for Antitumor Active Substance Research and Development, Department of Biochemistry and Molecular Biology, School of Basic Medicine, Guangdong Medical University, Zhanjiang 524023, China; ^6^Institute of Laboratory Medicine, School of Medical Technology, Guangdong Medical University, Dongguan 523808, China

## Abstract

Human gingiva-derived mesenchymal stem cells (GMSCs) are isolated from the gingival propria with promising regenerative, immunomodulatory, and anti-inflammatory properties. Recently, several studies, including ours, have found that GMSCs have the therapeutic potentials of nerve regeneration and skin disorders in various types such as the cell itself, cell-free conditioned medium, or extracellular vesicles (EVs). However, the mechanobiological behavior of GMSCs is closely related to the culture conditions. Therefore, the purpose of this study was to evaluate the function of human GMSCs on imiquimod- (IMQ-) induced murine psoriasis-like skin inflammation in two-dimensional (2D) and three-dimensional (3D) culture conditions. Here, we isolated and characterized GMSCs in 2D and 3D culture conditions and found that GMSCs in 2D and 3D infusion can significantly ameliorate the IMQ-induced murine psoriasis-like skin inflammation, reduce the levels of Th1- and Th17-related cytokines IFN-*γ*, TNF-*α*, IL-6, IL-17A, IL-17F, IL-21, and IL-22, and upregulate the percentage of spleen CD25^+^CD3^+^ T cells while downregulate the percentage of spleen IL-17^+^CD3^+^ T cells. In summary, our novel findings reveal that GMSCs in 2D and 3D infusion may possess therapeutic effects in the treatment of psoriasis.

## 1. Introduction

Mesenchymal stem cells (MSCs) are multilineage cells with self-renewal and multipotent differentiation, and immunomodulatory/anti-inflammatory properties play a vital role in tissue repair and regeneration [1, 2]. They are present in almost all tissues, including adipose tissue, bone marrow, umbilical cord, synovium, skeletal muscles, dental pulp, gingival, amnion, placenta, and skin [3–5]. MSCs commonly express similar cell surface molecules, such as CD29, CD44, CD73, CD90, and CD105, but typically lack hematopoietic cell markers, such as CD14, CD19, CD34, and CD45 [2, 6]. Human gingiva-derived mesenchymal stem cells (GMSCs) are isolated from the gingival propria with promising regenerative, immunomodulatory, and anti-inflammatory properties. Similar properties shared with other MSCs, including BMSCs, UMSCs and ADMSCs, GMSCs have several unique characteristics, specially their high proliferative capacity. In addition, GMSCs can keep MSC characteristics and show stable morphology and maintain telomerase activity under long-term culture conditions. Besides the differentiation potentials (osteocytes and adipocytes), GMSCs possess the potential to transdifferentiate into neural cells, endothelial cells, keratinocytes, and odontogenic cells under different induction conditions. Recently, several studies, including ours, have found that GMSCs have the therapeutic potentials of nerve regeneration and skin disorders in various types such as the cell itself, cell-free conditioned medium, or extracellular vesicles (EVs) [7–9]. A number of studies have found that the mechanobiological behavior of MSCs is closely related to the culture conditions. Most recently, some studies found that 3D spheroid GMSCs significantly increased the stem cell properties and therapeutic effects. In comparison to the 2D cultured GMSCs, 3D spheroid GMSCs showed enhanced multipotency and secreted an increased level of several chemokines and cytokines related with cell migration, proliferation, and angiogenesis; in vivo, 3D spheroid cultures of GMSCs improved mitigation of oral mucositis. The purpose of this study was to evaluate the function of human GMSCs on imiquimod- (IMQ-) induced murine psoriasis-like skin inflammation in two-dimensional (2D) and three-dimensional (3D) culture conditions.

Psoriasis is a T cell-mediated inflammatory autoimmune skin disease, with an imbalance between Th2 and Th1/Th17 cytokines [10]. MSCs could alleviate psoriasis skin lesions by suppressing the local levels of angiogenic and proinflammatory mediators and inhibiting activation and differentiation of DC-mediated CD4^+^ T cells [11, 12]. More recently, some studies showed that MSCs could be an effective treatment for psoriasis [11–17]. Up to date, there are nine clinical trials on MSC-based therapy of psoriasis. [2]. At present, this is due to the easy accessibility, genomic stability, the highly proliferative activity, less morbidity of harvesting, the potent immunomodulatory, and regenerative potentials, as well as well tolerated by all recipient hosts without any obvious systemic adverse effects [18]. GMSCs have become an attractive source of adult stem cells for regenerative therapy and tissue engineering.

However, whether GMSCs also has an effective treatment for psoriasis and whether this effect is related to the different mechanobiological behavior of GMSCs is unknown. To date, some studies have reported that aggregation of MSCs in 3D spheroid culture can significantly enhance their multipotent differentiation, anti-inflammatory properties, and angiogenic and tissue regenerative effects [19]. In this study, we isolated and characterized GMSCs in 2D and 3D culture conditions and found that GMSCs in 2D and 3D infusion can significantly ameliorate the IMQ-induced murine psoriasis-like skin inflammation, reduce the levels of Th1- and Th17-related cytokines IFN-*γ*, TNF-*α*, IL-6, IL-17A, IL-17F, IL-21, and IL-22, and upregulate the percentage of spleen CD25^+^CD3^+^ T cells while downregulate the percentage of spleen IL-17^+^CD3^+^ T cells. Our novel findings reveal that GMSCs in 2D and 3D infusion may possess therapeutic effects in the treatment of psoriasis.

## 2. Materials and Methods

### 2.1. GMSC Isolation and Culture

Approved by the Ethics Committee of Binhaiwan Central Hospital of Dongguan, human gingival tissue samples were collected from clinically healthy patients without history of periodontal disease. The isolation of human GMSCs was described previously [3]. The gingival tissues were treated aseptically and washed several times with phosphate buffered saline (PBS). Then, the tissues were minced into small fragments (1-3 mm^3^) and digested with collagenase IV (Sigma) solution at 37°C for 1 h and centrifuged at 1000 rpm for 5 min, and the supernatant was discarded. The cells were suspended in complete minimum essential medium *α* (*α*-MEM) containing 10% fetal bovine serum (FBS), penicillin (100 U/ml), and streptomycin (100 *μ*g/ml), then placed into 10 cm cell culture dish, and maintained at 37°C and 5% CO_2_ in a humidified incubator. The medium was refreshed every three days. After reaching 80% confluence, cells were digested with trypsin-EDTA solution (0.25%). Cells of passages 3-8 were used for the present experiments.

### 2.2. Multipotent Differentiation of GMSCs

#### 2.2.1. Osteogenic Differentiation

The GMSCs were seeded in 6-well plates (5 × 10^5^ cells/well) and incubated with *α*-MEM, allowed to adhere overnight, and replaced with osteogenic induction medium (Scien Cell) every 3 days. Four weeks later, in vitro mineralization was assayed by Alizarin Red S staining.

#### 2.2.2. Adipogenic Differentiation

As described above, the GMSCs were cultured in adipogenic differentiation medium (Scien Cell). The medium was refreshed every 3 days. Two weeks later, the cells were fixed and assessed by Oil Red O staining.

### 2.3. Spheroid Generation and Dissociation

GMSCs were seeded into ultralow attachment dishes (2 × 10^5^/ml) and incubated with complete *α*-MEM to allow 3D spheroid formation for up to 3 days. To acquire spheroid-derived GMSCs, spheroids were incubated with 0.25% trypsin at 37°C for 15 min while pipetting every 5 min; the single cells were collected by centrifugation.

### 2.4. Cell Proliferation Analysis

CCK-8 (Dojindo) assay was used to measure the viability of GMSCs in 2D (2D-GMSCs) and GMSCs in 3D (3D-GMSCs) culture conditions on a normal culture plate. Briefly, a total of 1000 cells per well were cultured in five replicate wells in a 96-well plate. Then, 10 *μ*l CCK-8 reagent was added to each well and cultured for 2 h. The absorbance was measured at 450 nm using a microplate reader. We performed this assay on day 1, day 3, day 5, and day 7.

### 2.5. Flow Cytometry

GMSCs were collected and suspended in cell staining buffer (0.5% BSA in PBS with 2 mM EDTA) followed by incubation with CD14 (PE), CD19 (PerCP-Cy5.5), CD29 (APC), CD34 (PE), CD44 (FITC), CD45 (PE), CD73 (PE), CD90 (FITC), and CD105 (PE) antibodies (Biolegend) in the dark at room temperature for 30 min. For intracellular staining, splenocytes from mice were first stained with CD3 (APC/Cy7), CD4 (FITC), and CD25 (APC) antibodies (Biolegend) and then fixed, permeabilized, and stained intracellularly for IL-4 (PE/Cy7) and IL-17 (PE). After staining, cells were washed twice with PBS and submitted to flow cytometric analysis (BD). Data were analyzed using the FlowJo 7.6 software.

### 2.6. Cytokine Analysis

For tissue cytokine analysis, the protein was extracted from skin tissue, then homogenate was centrifuged at 10,000 × g for 10 min at 4°C, and supernatant was collected. Protein concentration was determined using BCA protein Assay Kit. Cytokines (IL-6, IL-10, IL-17A, IL-17F, IL-21, IL-22, TNF-*α*, and IFN-*γ*) in the serum and skin lysate were measured by the LEGENDplex Multi-Analyte Flow Assay kit (Biolegend, 740749) following the manufacturer's instructions. Briefly, 25 *μ*l of the standard, serum or skin lysate, and buffer solutions was added to the wells. To each well, 25 *μ*l of mixed beads was added. Then, the plate was covered with a plate sealer and shook at 500 rpm for 2 h at room temperature. After 2 washes, 25 *μ*l of biotinylated detection antibodies was added to each well. The plate was then covered with a plate sealer and shook at 500 rpm for 1 h at room temperature. Subsequently, 25 *μ*l of Streptavidin-phycoerythrin was added to each well, and the plate was covered with a plate sealer and shook at 500 rpm for 30 min at room temperature. After 2 washes, the samples were tested on a flow cytometer. The results were analyzed using the LEGENDplex data analysis software. The concentration of each analyte was quantified in pictograms per milliliter.

### 2.7. RNA Sequencing

The cells were collected and lysed by TRIzol, and total RNA was extracted according to the manufacturer's instructions (Invitrogen). RNA was quantified using Nanodrop spectrophotometer (Thermo Scientific). RNA sequencing was carried out by the Guangdong Longsee Biomedical Co., Ltd following standard protocols. Standard bioinformatics analysis was performed by the Guangdong Longsee Biomedical Co., Ltd.

### 2.8. Animals

Female C57BL/6 mice weighing 20 g (8 weeks old) were purchased from the Guangdong Medical Laboratory Animal Center (Foshan, China). Animal experiments in the study were approved by the Animal Experimental Ethics Committee of Guangdong Medical University in compliance with the National Guidelines for the Care and Use of Animals. Mice were group-housed in polycarbonate cages in the animal facilities with controlled temperature (23°C ± 2°C), 40%-65% humidity, and a 12-hour light/dark cycle. Mice were acclimatized for at least 1 week before the study, fed with a standard laboratory diet, and allowed free access to drinking water.

### 2.9. Establishment and Treatment of IMQ-Induced Murine Psoriasis-Like Skin Inflammation

Fifteen mice were randomly divided into three groups, with five mice in each, as follows: the IMQ control group, 2D-GMSC treatment group, and 3D-GMSC treatment group. On the day 0, the backs of mice were shaved using depilatory machine and cream. 5% imiquimod (IMQ) cream (Sichuan Mingxin) was used to induce psoriasis-like skin inflammation with a daily dose of 62.5 mg from day 1 to day 7, consecutively.

The effects of GMSCs were tested by administration of 2 × 10^6^ 2D-GMSCs or 3D-GMSCs in 200 *μ*l PBS via the mouse tail vein on day 1 and day 4 consecutive IMQ treat. The IMQ control group received an intravenous injection of 200 *μ*l PBS via the tail vein on day 1 and day 4. The severity of the inflammation of the psoriatic skin was assessed using the Psoriasis Area Severity Index (PASI). The degree of erythema, scaling, or thickening was each scored on a scale from 0 to 4, as follows: 0, none; 1, slight; 2, moderate; 3, marked; and 4, severe. The cumulative PASI scores (erythema+scaling+thickening) were calculated to reflex the severity of inflammation. All mice were sacrificed on day 8, and blood, spleen, and skin samples were collected for further studies.

### 2.10. Histology and Immunohistochemical Analysis

The back skin of all the mice was fixed with 10% Paraformaldehyde solution (PFA). For histological study, paraffin-embedded sections were stained with hematoxylin-eosin (HE) staining. For immunohistochemical studies, the paraffin-embedded sections (4 *μ*m) were deparaffinized with xylene, rehydrated with graded ethanol, and heated in 10 mmol/L sodium citrate buffer (pH 6.0) for antigen retrieval. After blocking with 2.5% goat serum in PBS, the sections were incubated overnight at 4°C with primary antibodies (TNF-*α*, IL-6, IFN-*γ*, and IL-17A) and then detected using the universal immunoperoxidase ABC kit. All the sections were counterstained with hematoxylin. Images were captured using a light microscope (Olympus).

### 2.11. Statistical Analysis

All data are presented as mean ± standard deviation (SD) from at least three independent experiments. Differences between experimental and control groups were analyzed by a two-tailed unpaired Student's *t*-test using the GraphPad Prism 7 software. A value of less than 0.05 was considered statistically significant.

## 3. Results

### 3.1. Characterization of GMSCs under 2D and 3D Culture Conditions

**Figure 1:**
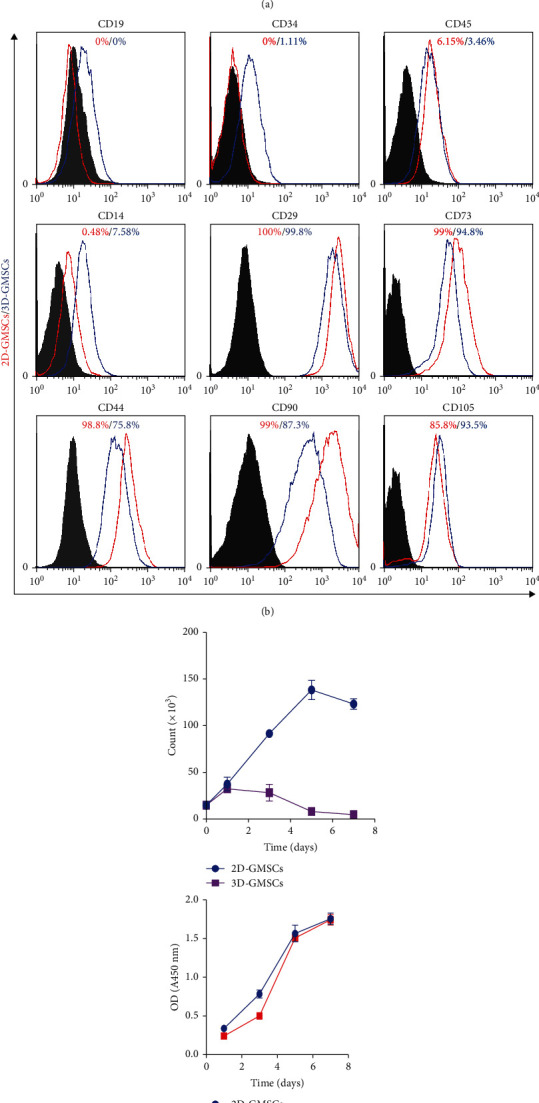
Characterization of GMSCs. (a) Representative images of 2D cultured GMSCs (A). Morphology of GMSCs cultured on a low attachment culture dish (B). Representative images of adipogenesis (C) and osteogenesis (D). (b) Flow cytometric analysis of surface markers in GMSCs under 2D or 3D conditions. (c) Proliferation assay for GMSCs cultured on a low attachment culture dish.

**Figure 2:**
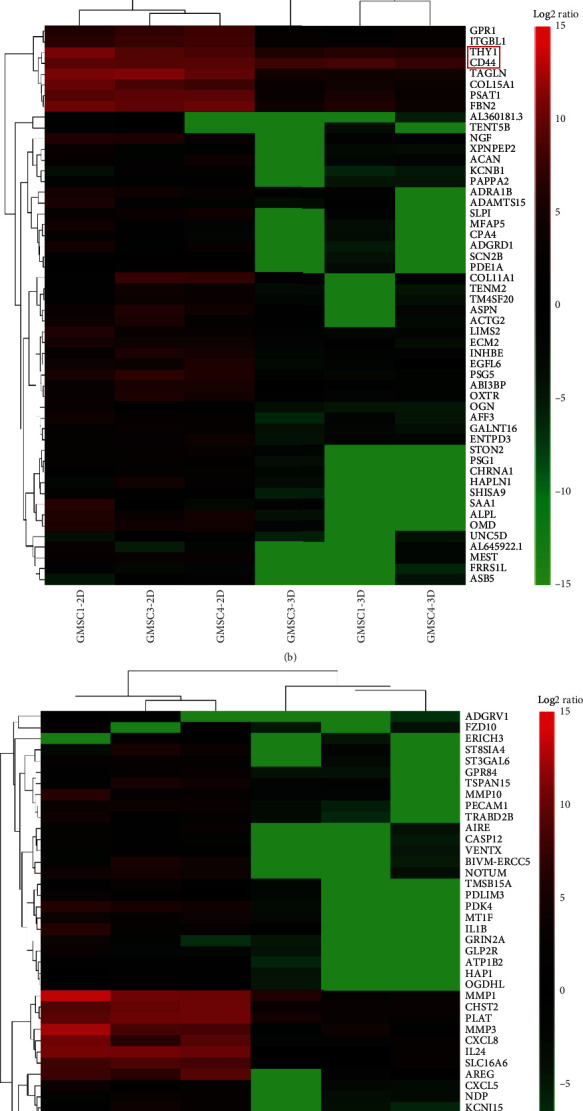
The 3D culture of GMSCs caused significant alterations in gene expression. (a) The difference of gene expression between 2D-GMSCs and 3D-GMSCs was analyzed by RNA sequencing. (b, c) The representative global view of gene expression changes between 2D-GMSCs and 3D-GMSCs. (d) Gene ontology analysis of differentially expressed genes. BP: biological process; CC: cellular component; MF: molecular function.

As shown in Figure 1(a), we have successfully isolated GMSCs from gingival tissues and the cells exhibited a spindle-like morphology in 2D culture conditions (Figure 1(a), A) and spontaneously aggregated into 3D spheroids under growth condition of ultralow attachment (Figure 1(a), B). The results of osteogenic and adipogenic differentiation experiments demonstrated that GMSCs have multiple differentiation capabilities (Figure 1(a), C and D). Flow cytometry analysis showed that GMSC cells were strongly positive for MSC markers CD29, CD44, CD73, CD90, and CD105, but negative for hematopoietic cell markers CD14, CD19, CD34, and CD45 (Figure 1(b)). These results were consistent with our previous studies [3]. The results of flow cytometry analysis showed that 3D-GMSCs also negatively expressed CD14, CD19, CD34, and CD45 (Figure 1(b)). And the expression of CD29, CD44, CD73, CD90, and CD105 was decreased, compared with 2D-GMSCs (Figure 1(b)). The cell proliferation results showed that the proliferation rate of GMSCs under 3D culture conditions (3D-GMSCs) was reduced compared with GMSCs under 2D culture conditions (2D-GMSCs) (Figure 1(c)). It is worth noting that when the 3D-GMSCs were replanted in the 2D culture conditions, the cell proliferation rate of replanted 3D-GMSCs (RA-3D-GMSCs) was restored, similar to 2D-GMSCs (Figure 1(c)). The RNA sequencing results showed that among the 13844 screened genes, altogether 1312 genes were significantly upregulated and 1022 genes were significantly downregulated in 3D cultured GMSCs compared with 2D cultured GMSCs (|log2(fold of gene expression change)| ≥ 1) (Figures 2(a)–2(c)). Gene ontology analysis of differentially expressed genes was performed using the Database for Annotation, Visualization and Integrated Discovery (DAVID) online tool. The results of the GO analysis found that alterations of biological processes (BPs) in the differentially expressed genes were significantly changed in the signal transduction, inflammatory response and oxidation-reduction process (Figure 2(d)). The differentially expressed genes' alterations in the cell component (CC) were mainly located in plasma membrane, extracellular exosome, and extracellular region (Figure 2(d)). The alterations in gene functionality at the molecular level (MF) were mainly associated with extracellular matrix structural constituent, collagen binding and growth factor activity (Figure 2(d)).

### 3.2. GMSCs Attenuate the Symptoms of IMQ-Induced Murine Psoriasis-Like Skin Inflammation under 2D and 3D Culture Conditions

**Figure 3:**
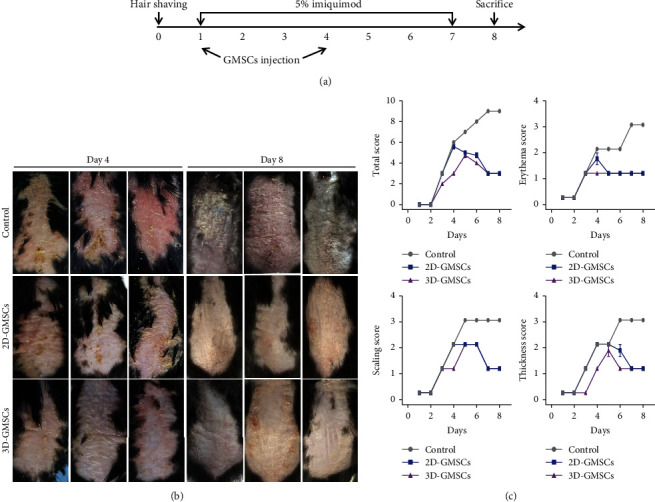
2D-GMSCs and 3D-GMSCs significantly ameliorated psoriatic symptoms in IMQ-induced mice. MSC infusion ameliorated psoriatic symptoms in IMQ-induced mice. (a) Experimental protocol showing treatment regimens using 2D-GMSCs and 3D-GMSCs in psoriatic mice. (b) Typical presentation of the mouse back skin from IMQ, 2D-GMSCs, and 3D-GMSCs group on day 4 and day 8 was, respectively, shown. (c) Different levels of erythema, scales, thickness of back skin, and total score were scored daily.

To determine whether GMSCs also have an effective treatment for psoriasis, and whether this effect is related to the different mechanobiological behavior of GMSCs, we established a murine model of IMQ-induced psoriasis-like skin inflammation that was injected with 2D-GMSCs and 3D-GMSCs. The intervention strategy is shown in Figure 3(a). The morphological observation of the back skin is shown in Figure 3(b). IMQ control group mice exhibited the most serious symptoms of erythema, scaling, and thickness, which continuously increased in severity up to the end of IMQ application on day 7. However, 2D-GMSC and 3D-GMSC treatment significantly reduced the severity of skin lesions in mice. We also assessed the severity of psoriasis-like skin and total scores of skin lesions on days 1-8 via PASI scoring system. The PASI scores were gradually increased in IMQ control group mice. Interestingly, 2D-GMSC and 3D-GMSC treatment significantly decreased the PASI score (Figure 3(c)). These results suggest that GMSCs attenuate the symptoms of IMQ-induced murine psoriasis-like skin inflammation under 2D and 3D culture conditions.

### 3.3. GMSCs Attenuate the Skin Inflammation of IMQ-Induced Murine Psoriasis-Like Skin Inflammation under 2D and 3D Culture Conditions

**Figure 4:**
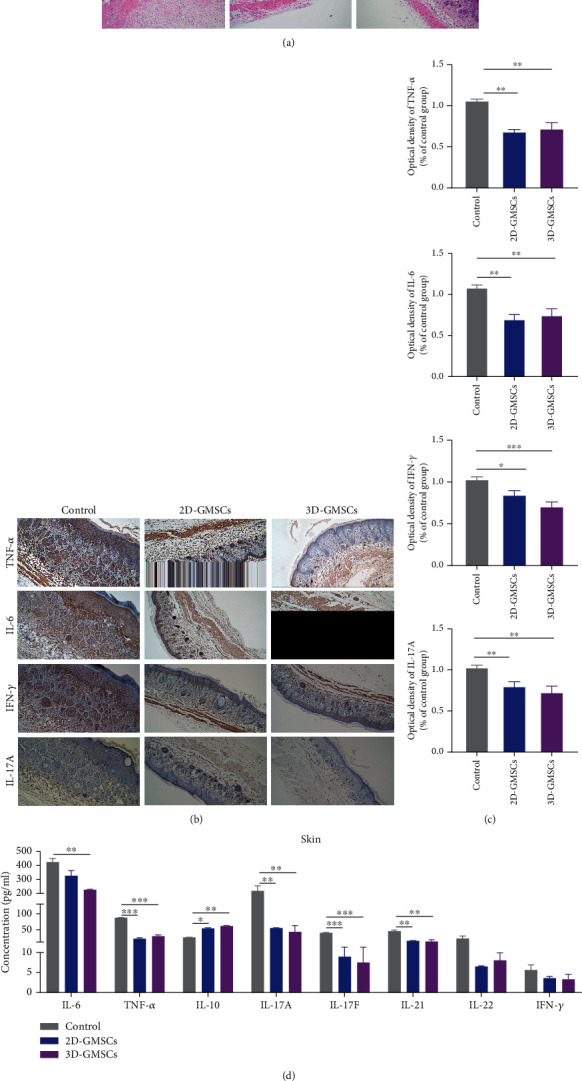
2D-GMSCs and 3D-GMSCs significantly inhibited the skin inflammatory response in IMQ-induced mice. (a) Representative HE staining of mouse back skin. (b) Immunohistochemical studies were performed using antibodies for mouse TNF-*α*, IL-6, IFN-*γ*, and IL-17A. (c) The relative expression levels of TNF-*α*, IL-6, IFN-*γ*, and IL-17A were analyzed by pathological score (PS). ^∗^*p* < 0.05; ^∗∗^*p* < 0.01; ^∗∗∗^*p* < 0.001. (d) The protein expression level of inflammatory cytokines in the skin tissue lysate evaluated via multiplex LEGENDplex analysis. ^∗^*p* < 0.05; ^∗∗^*p* < 0.01; ^∗∗∗^*p* < 0.001.

HE staining further confirmed that 2D-GMSCs and 3D-GMSCs significantly decreased epidermal thicknesses (Figure 4(a)). Immunohistochemical studies demonstrated that treatment with 2D-GMSCs and 3D-GMSCs inhibited the expression of TNF-*α*, IL-6, IFN-*γ*, and IL-17A (Figures 4(b) and 4(c)). We also analyzed the levels of IL-6, IL-17A, IL-17F, IL-21, IL-22, TNF-*α*, and IFN-*γ* in the skin lysate. 2D-GMSC and 3D-GMSC treatment significantly reduced the levels of IL-6, TNF-*α*, IL-17A, IL-17F, and IL-21 and increased the level of IL-10 in the skin compared to that in control (Figure 4(d)). These results suggest that GMSCs significantly reduced the proinflammatory response, while upregulating the anti-inflammatory response of IMQ-induced murine psoriasis-like skin inflammation under 2D and 3D culture conditions.

### 3.4. GMSCs Strongly Influence the Expression of Inflammatory Mediators and Spleen CD25^+^CD3^+^ and IL-17^+^CD3^+^ T Cell Responses of IMQ-Induced Murine Psoriasis-Like Skin Inflammation under 2D and 3D Culture Conditions

**Figure 5:**
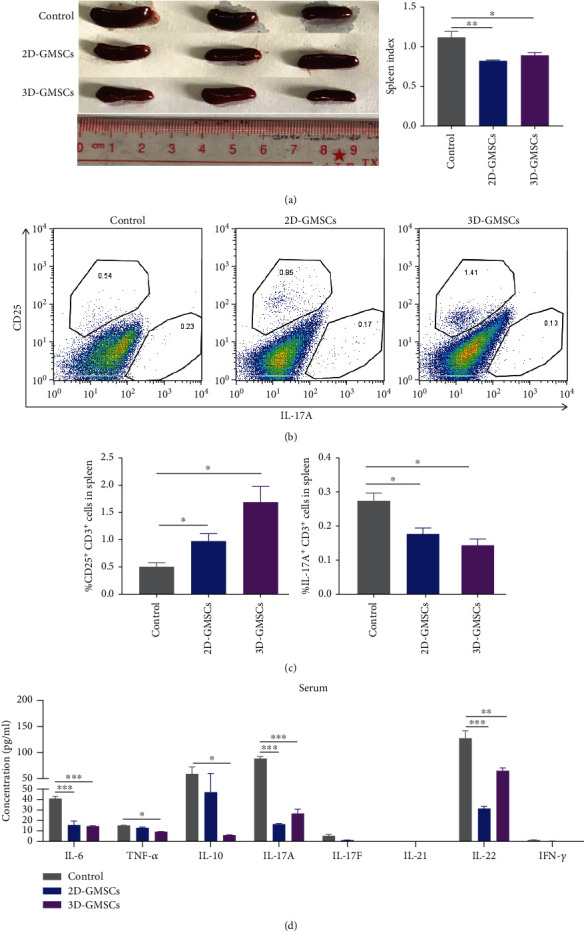
GMSCs strongly influence the expression of serum inflammatory mediators and spleen CD25^+^CD3^+^ and IL-17^+^CD3^+^ T cell responses. (a) Representative photographs of spleen and the ratio of spleen weight to bodyweight in different groups. ^∗^*p* < 0.05; ^∗∗^*p* < 0.01. (b) Representative flow cytometry staining for CD25 and IL-17A in CD3^+^ T cells from mouse spleen. (c) Quantification of percentage of CD25^+^CD3^+^ T cells and IL-17A^+^CD3^+^ T cells from flow cytometry data. ^∗^*p* < 0.05. (d) The protein expression level of inflammatory cytokines in the serum evaluated via multiplex LEGENDplex analysis. ^∗^*p* < 0.05; ^∗∗^*p* < 0.01; ^∗∗∗^*p* < 0.001.

Additionally, the mouse spleen volumes and spleen/body weight ratio in the 2D-GMSC and 3D-GMSC group were reduced compared to the IMQ control group (Figure 5(a)). Furthermore, we determined the percentage of Th17 and Treg cells in the spleen of mice, and splenocytes from all the mice were stained for CD3, CD25, and IL-17A. After 2D-GMSC and 3D-GMSC treatment, the percentage of IL-17A^+^CD3^+^ T cells was notably reduced, while the percentage of CD25^+^CD3^+^ T cells was significantly increased compared with the IMQ control group (Figures 5(b) and 5(c)). These data suggested that GMSCs could modulate spleen Th17 and Treg responses of IMQ-induced murine psoriasis-like skin inflammation under 2D and 3D culture conditions. We also analyzed the levels of IL-6, IL-10, IL-17A, IL-17F, IL-21, IL-22, TNF-*α*, and IFN-*γ* in the serum. As shown in Figure 5(d), the levels of IL-6, TNF-*α*, IL-10, IL-17A, and IL-22 in the serum of mice were significantly decreased in the 2D-GMSC and 3D-GMSC group compared with the IMQ control group (Figure 5(d)). These data suggested that GMSCs could modulate the expression of serum inflammatory mediators of IMQ-induced murine psoriasis-like skin inflammation under 2D and 3D culture conditions.

## 4. Discussion

In the present study, we successfully isolated GMSCs from human gingival tissue, and these cells showed osteogenic and adipogenic differentiation capabilities. Additionally, the isolated GMSCs positively expressed CD29, CD44, CD73, CD90, and CD105 but did not express hematopoietic stem cell markers such as CD14, CD19, CD34, and CD45. Traditionally, 2D culture conditions have been used as a standard technique for in vitro expansion of MSCs. Compared with 2D cell culture, 3D culture was regarded as more physiological with the characteristics better reserved [20]. In our study, we firstly observed the growth of GMSCs under 3D culture conditions. They spontaneously aggregated into spheroids under condition of ultralow attachment. In addition, the lower proliferation rate showed that 3D culture affected the growth characteristics of GMSCs. Next, the RNA sequencing analysis showed that a lot of genes involved in ossification, cytokine, mesenchymal cell differentiation, and chemotaxis were differentially regulated in 3D-GMSCs compared with 2D-GMSCs. Some previous studies on UC-MSCs and human bone marrow- and adipose tissue-derived MSCs revealed that 3D culture caused a significant upregulation of angiogenetic genes and promoted expression of proinflammatory and anti-inflammatory genes at the transcription level [21].

After RNA sequencing analysis, we focused on the effect of 3D culture on the immunophenotype of 3D-GMSCs through flow cytometry. Similar MSC marker expression was observed in 3D-GMSCs and 2D-GMSCs, whereas interestingly, the expression of CD44 and CD90 was downregulated in 3D-GMSCs. CD73 (Ecto-5′-nucleotidase, e-5′NT), a rate-limiting enzyme in the extracellular metabolism of ATP, can convert ATP to immunosuppressive adenosine; therefore, it is considered an important mediator of immunity [22]. Some studies demonstrated that CD73 expression was decreased in 3D spheroid-derived MSCs compared to the 2D cultured MSCs [23, 24]. Consistently, our results also showed the CD73 expression was downregulated at protein level in 3D spheroid-derived MSCs. CD90 (Thy1) is a glycophosphatidylinositol-anchored membrane protein highly expressed by MSCs [25]. According to reports, in vivo and in vitro, periosteum-derived cells sorted with CD90 have higher osteogenic potential than unsorted cells [26]. Recently, a study showed that compared with MSCs from wild-type mice, the osteogenic differentiation ability of MSCs from Thy1 knockout mice was reduced [27]. Together, these findings showed that CD90 was related with osteogenic differentiation. Most recently, studies have shown that 3D spheroids from AMSCs and WJ-MSCs showed higher expression of the osteogenic markers Runx2, osteopontin, and ALP at mRNA level than 2D cultured cells [28, 29]. Our present study showed that 3D-GMSCs exerted lower expression of the CD90 at mRNA and protein level than 2D-GMSCs.

Psoriasis is a chronic, Th1/Th17-mediated inflammatory disease, which is related to the excessive proliferation and differentiation of abnormal keratinocytes, resulting in erythema, thickness, and scaly plaques [30]. It affects an estimated 125 million people worldwide [31]. Initially, Th1 cells and the cytokines produced by these cells, such as TNF-*α* and IFN-*γ*, were associated with psoriasis [32]. Numerous studies showed that Th17 cells and their inflammatory mediators play an important role in the pathogenesis of psoriasis [33]. Th17 cytokines, such as IL-6, IL-17A, IL-17F, IL-21, and IL22, act on keratinocytes, leading to their activation and overproliferation [34, 35]. The activated keratinocytes in turn promote the recruitment of inflammatory cells [34, 35]. The current clinical treatment of psoriasis completely involves topical drugs, including vitamin D3 analogues, topical corticosteroids, calcineurin inhibitors, keratolytics, and biologics that inhibit TNF-*α*, IL-12, IL-13, IL-17, and IL-23 [31].

IMQ is an agonist of Toll-like receptors (TLR) 7 and 8, used to treat warts, and has been widely used to induce psoriasis-like skin inflammation [36]. Several studies have shown that MSCs and exosomes derived from MSCs can effectively improve psoriasis-like skin lesions in mouse models [37–39]. Here, this is the first study to investigate the therapeutic potential of 2D cultured GMSCs and spheroid-derived GMSCs in psoriasis-like lesions induced by IMQ administration in a mouse model. Our results showed that GMSCs reduced erythema, skin thickness, and scaling exerted protective effects against psoriasis-like skin inflammation induced by IMQ under 2D and 3D culture conditions. Furthermore, HE staining confirmed that 2D-GMSCs and 3D-GMSCs could prevent the proliferation and abnormal differentiation of keratinocytes. The spleen is a major organ of the immune system and secreting a variety of immune-active cytokines; therefore, it plays an important role in immune activities. In this study, we found that 2D-GMSCs and 3D-GMSCs significantly inhibited the ratio of spleen to body weight, indicating that GMSCs under 2D and 3D culture conditions can regulate the inflammatory immune cells of the spleen to produce an inflammatory immune response with a systemic antipsoriatic effect. GMSCs cultured in 3D and 2D have similar effects. We found that 2D-GMSCs and 3D-GMSCs can inhibit IMQ-induced inflammation in psoriasis-like mouse models. 2D-GMSCs and 3D-GMSCs significantly reduced the serum levels of Th1 cytokines (TNF-*α* and IL-6), Th17 cytokines (IL-17A and IL-22), and IL-10, which means intravenous injection 2D-GMSCs and 3D-GMSCs inhibit IMQ-induced inflammation. Consistently, these results were observed in the skin; conversely, the level of IL-10 was increased. These results indicate that 2D-GMSCs and 3D-GMSCs inhibit IMQ-induced Th1/Th17 cytokine and psoriasis skin changes. However, further research is needed to determine the exact molecular mechanism of GMSC's anti-inflammatory effects. Finally, we measured the percentage of Treg and Th17 in the mouse spleen. We found that the ratio of Treg cells increased after treatment with 2D-GMSCs and 3D-GMSCs, while the ratio of Th17 cells decreased, showing that 2D-GMSCs and 3D-GMSCs exert immunomodulatory and anti-inflammatory properties. In summary, we revealed that transplantation of 2D-GMSCs and 3D-GMSCs has therapeutic potential for the treatment of psoriasis-like skin lesions.

## Data Availability

All of the data used to support the findings of this study are included within the article.
